# The changing patterns of dispensing branded and generic drugs for the treatment of gastroesophageal reflux disease between 2006 and 2011 in Japan: a retrospective cohort study

**DOI:** 10.1186/s12913-015-0734-2

**Published:** 2015-02-27

**Authors:** Kyoko Murata, Shiro Hinotsu, Shota Hamada, Yasumasa Ezoe, Manabu Muto, Koji Kawakami

**Affiliations:** Department of Pharmacoepidemiology, Graduate School of Medicine and Public Health, Kyoto University, Yoshidakonoe-cho, Sakyo-ku, Kyoto, 606-8501 Japan; Center for Innovative Clinical Medicine, Okayama University Hospital, Okayama, Japan; Department of Therapeutic Oncology, Kyoto University Hospital, Kyoto, Japan

**Keywords:** Generic drug, Branded drug, Drug utilization research, Dispensing pattern, Gastroesophageal reflux disease

## Abstract

**Background:**

Despite rising healthcare costs, generic drugs are less frequently dispensed in Japan compared with other developed countries. This study aimed to describe changes in dispensing of branded and generic drugs and to explore possible factors that promote the use of generic drugs.

**Methods:**

We conducted a retrospective cohort study using a Japanese medical and pharmacy claims database. All proton pump inhibitors (PPIs) and histamine H_2_-receptor antagonists (H_2_RAs) with indications for gastroesophageal reflux disease (GERD) described on Japanese labels were included. Patterns of dispensing branded and generic drugs for the treatment of GERD between 2006 and 2011 were analyzed. Multivariate logistic regression was applied to investigate factors associated with receiving generic drugs.

**Results:**

The study cohort included 14,590 patients (male: 50.2%, mean age: 43.1 years). Branded drugs for GERD were still frequently dispensed despite an increase in the share of generic drugs. Only 4.3% of patients who initially received branded drugs switched to generic drugs. The percentage of patients who received only generic drugs increased over time (6.5% to 22.1%). The frequency of generic drug dispensing was the highest in the setting where both prescription and dispensing were implemented in clinics (43.3%), while the lowest in the setting where both prescription and dispensing were implemented in hospitals (11.5%). Factors associated with receiving generic drugs included year of dispensing (adjusted OR 2.22, 95% CI 1.94 to 2.55 for 2009–11 v 2006–8), prescription and dispensing setting (OR 1.81, 95% CI 1.44 to 2.26 for prescription in hospitals and dispensing in community pharmacies; OR 2.21, 95% CI 1.80 to 2.72 for prescription in clinics and dispensing in community pharmacies; and OR 4.55, 95% CI 3.68 to 5.62 for prescription and dispensing in clinics v prescription and dispensing in hospitals) and H_2_RAs (OR 1.64, 95% CI 1.49 to 1.81 compared to PPIs).

**Conclusions:**

The share of generic drugs for the treatment of GERD increased over time although branded drugs for GERD were still dispensed frequently. The use of generic drugs for GERD was influenced not only by government policies but also by changes in treatment approach and the setting of prescription and dispensing.

## Background

Generic drugs can help reduce healthcare costs and patients’ co-payments with replacement of more expensive branded drugs including the same active molecule which have expired the patents. To date, several policies and measures have been enacted to promote the use of generic drugs by the Ministry of Health, Labour and Welfare in Japan, with some moderate success seen in increasing the share of generic drugs (18.7% in 2007 to 22.8% in 2011 in volume share) [1-5]. The measures included, for example, modification of the prescription form and incentive for generic drug prescribing and dispensing to medical institutions and pharmacies. However, despite the increase in total healthcare costs being a concern in Japan, the share of generic drugs is still much lower than in other developed countries [6,7].

The use of commercially available generic drugs is not mandatory in Japan. At present, physicians, pharmacists and patients are all involved in the process of selecting branded or generic drugs. For example, when physicians want their patients to use branded drugs, they can instruct pharmacists not to dispense generic drugs [8]. Conversely, even when physicians permit patients to use generic drugs, pharmacists and patients can refuse generic drugs and patients can receive branded drugs instead. According to recent surveys, approximately 70 to 90% of the general public or patients were aware of generic drugs and only 10% of the general public or patients, physicians, and pharmacists viewed them negatively [9-11]. However, concerns remained over the quality and stable supply of generic drugs in Japan [9].

The utilization of generic drugs has been investigated in terms of therapeutic areas rather than specific diseases or drug classes in Japan [12,13]. In the Netherlands, more patients treated with omeprazole switched to other branded molecules of proton pump inhibitors (PPIs) after the patent expiration of omeprazole [14]. In the United States, generic forms of statins, such as pravastatin and simvastatin, substituted for branded atorvastatin from the observation of share trend within statins [15]. Generic anti-epileptic drugs were not admissible to patients in comparison to other long-term therapies with generic forms of anti-hyperlipidemics and anti-depressants [16]. Such detailed analyses of drug utilization would give effective implications for promotion of generic drugs.

Gastroesophageal reflux disease (GERD) is one of the most common gastrointestinal disorders; the prevalence of patients experiencing at least weekly reflux symptoms is reported as 6.5 to 9.5% in Japan [17-19]. Most patients with GERD are treated with acid-suppressive drugs in outpatient settings. PPIs are the first-line for the treatment of GERD but histamine H_2_-receptor antagonists (H_2_RAs) can also be used [20]. In this study, PPIs and H_2_RAs for GERD were considered appropriate for investigating the utilization and the impact of generic drugs, as both branded and generic drugs were commercially available during the study period. We analyzed the changing patterns of dispensing of branded and generic drugs for the treatment of GERD.

## Methods

### Data source

This was a retrospective cohort study using a medical and pharmacy claims database established by the Japan Medical Data Center Co., Ltd. (JMDC, Tokyo, Japan) [21,22]. The Japanese healthcare system is characterized by a universal health care coverage provided by employee-based or community-based plans and free access to clinics and hospitals. The claims database for the study, which comprises data from multiple employee-based insurances, was launched in 2005 with approximately 320,000 population and, as of 2010, includes approximately 1 million population. The database includes data on patient demographics such as encrypted personal identifiers, year of birth, sex, medical practices such as diagnosis, treatments, drugs dispensed, test orders, and type of medical institution and pharmacy and type of service (inpatient or outpatient). Diagnosis was defined according to the 10th revision of the International Classification of Diseases (ICD-10) [23] and standardized diagnoses provided by the Medical Information System Development Center (MEDIS-DC) [24].

### Prescription and dispensing procedure

In Japan, patients receive prescribed drugs via an in-house or community pharmacy. To date, it was shown that the share of generic drugs differed by prescription and dispensing procedure (ie, prescribing and dispensing separated or integrated) and larger hospitals used less generic drugs [25]. Therefore, we classified the prescription and dispensing process into four patterns according to the combinations of medical institution (hospital or clinic) and pharmacy (in-house or community) as follows: 1) prescription and dispensing in a hospital (in-hospital dispensing), 2) prescription and dispensing in a clinic (in-clinic dispensing), 3) prescription in a hospital, but dispensing in a community pharmacy (out-hospital dispensing), and 4) prescription in a clinic, but dispensing in a community pharmacy (out-clinic dispensing). Clinics are defined as medical institutions with less than 20 beds, whereas hospitals are defined as medical institutions with 20 or more beds in Japan.

### Patients

We used claims data from April 2006 to December 2011 to identify new patients with GERD. We defined new patients as those who did not have previous diagnoses of GERD in the last six months. Patients eligible for the study were 18 to 74 years old at the time of the initial dispensing of PPIs or H_2_RAs for the treatment of GERD in an outpatient setting. The initial diagnosis was limited to reflux esophagitis, GERD, or non-erosive GERD, and from the second diagnosis, intractable reflux esophagitis and intractable reflux esophagitis requiring maintenance treatment were also included according to the MEDIS-DC. PPIs or H_2_RAs dispensed more than six months after the completion of a series of initial treatments for new patients were regarded as treatments for the relapse of GERD and excluded in the analysis.

The following exclusion criteria were set to exclude non-GERD patients and to investigate the conditions at the initial dispensing and switching of branded and generic drugs clearly: 1) patients who were diagnosed with GERD but received no PPIs or H_2_RAs, 2) patients who could not be followed up for more than six months after the initial diagnosis, 3) patients who received drugs to be taken “as needed” at the initial dispensing, 4) dosing duration of the initial dispensing <7 or >56 days, 5) patients who received oral medicines of non-steroidal anti-inflammatory drugs or corticosteroids with PPIs or H_2_RAs at the initial dispensing, and 6) patients who received more than one PPIs and/or H_2_RAs at the initial dispensing or at switching between branded and generic drugs.

### Drugs of interest

All branded and generic drugs with indications for GERD described on Japanese labels were included in the study. These were omeprazole, lansoprazole, rabeprazole, and esomeprazole as PPIs, and famotidine, ranitidine, cimetidine, roxatidine, nizatidine, and lafutidine as H_2_RAs. Generic rabeprazole was marketed in November 2010, and branded esomeprazole was marketed in September 2011. No generic lafutidine was available during the study period. Both branded and generic drugs were commercially available for the other molecules throughout the study period.

### Analysis

The dispensing patterns and time trends of drug utilization were examined. Eligible patients were divided into two groups based on whether branded or generic drugs were initially dispensed, then into four subgroups based on switching patterns and frequency of dispensing (Table 1). Age, sex, and drugs dispensed (PPIs or H_2_RAs) were summarized using descriptive statistics for all patients and for each group. Patients stratified by the eight dispensing patterns were presented in chronological order when they were initially dispensed to analyze time trends of dispensing patterns. The time trends of frequencies of generic drug dispensing were investigated according to the four types of prescription and dispensing settings based on type of medical institution and pharmacy. The volume and value shares by branded or generic, PPIs or H_2_RAs, and individual molecules were calculated over time. The shares were calculated based on IMS’s methods of standard unit [26,27]. Conditions at switching between branded and generic drugs were analyzed in detail in patients who experienced switching once. Multivariate logistic regression was performed to investigate factors associated with receiving generic drugs using data at the initial dispensing. Covariates included sex, age, year of dispensing, setting of prescription and dispensing, and drugs dispensed. Adjusted odds ratios (OR) and 95% confidence intervals (CI) were reported as well as P values. Analysis was performed with IBM SPSS Statistics Version 20 and 21. The protocol of this study was approved by ethics committees at both Kyoto University Graduate School and Faculty of Medicine and the JMDC which has a licence for secondary use of the claims data.

**Table 1:** **Dispensing patterns of branded and generic drugs**

**Dispensing pattern**	**Patients n (%)**	**Male n (%)**	**Age (years) mean ± SD**	**Initial dispensing**		**All dispensing**	
				**% of PPIs**	**% of H**_**2**_ **RAs**	**% of PPIs**	**% of H**_**2**_ **RAs**
1. Branded (once)	6328 (43.4)	3256 (51.5)	41.3 ± 11.8	82.0	18.0	n/a	n/a
2. Generic (once)	1385 (9.5)	644 (46.5)	40.7 ± 11.8	70.8	29.2	n/a	n/a
3. Branded (≥twice)	4979 (34.1)	2476 (49.7)	45.0 ± 12.3	79.7	20.3	79.0	21.0
4. Generic (≥twice)	1000 (6.9)	476 (47.6)	45.3 ± 12.5	76.6	23.4	77.7	22.3
5. Branded to Generic (switched once)	486 (3.3)	261 (53.7)	47.3 ± 12.1	82.9	17.1	80.5	19.5
6. Generic to Branded (switched once)	198 (1.4)	106 (53.5)	43.9 ± 12.4	58.6	41.4	78.7	21.3
7. Started with Branded (switched multiple times)	142 (1.0)	62 (43.7)	46.3 ± 12.6	81.0	19.0	81.5	18.5
8. Started with Generic (switched multiple times)	72 (0.5)	42 (58.3)	47.5 ± 12.7	62.5	37.5	76.5	23.5

SD: standard deviation, PPIs: proton pump inhibitors, H_2_RAs: histamine H_2_-receptor antagonists.

## Results

### Dispensing patterns

A total of 14,590 patients (male: 50.2%) were included, and mean (±SD) age at the initial dispensing was 43.1 (±12.2) years. The percentage of patients who received PPIs or H_2_RAs only once was 52.9%. At the initial dispensing, 81.8% of patients received branded drugs, and 79.4% of patients received PPIs. Analysis of eight dispensing patterns showed that the majority of patients received only branded drugs; once (43.4%, pattern 1) and multiple times (34.1%, pattern 3) (Table 1). In contrast, the percentage of patients who received only generic drugs was 16.3%; once (9.5%, pattern 2) and multiple times (6.9%, pattern 4). Among the patients receiving PPIs and/or H_2_RAs multiple times, only a small percentage switched between branded and generic drugs. At the initial dispensing, patients who received branded drugs (patterns 1, 3, 5, and 7) were more likely to receive PPIs than those who received generic drugs (compared with patterns 2, 4, 6, and 8, respectively). Patients switching from generic to branded drugs were more likely to receive PPIs after the switch (patterns 6 and 8).

### Changes of dispensing patterns over time

**Figure 1:**
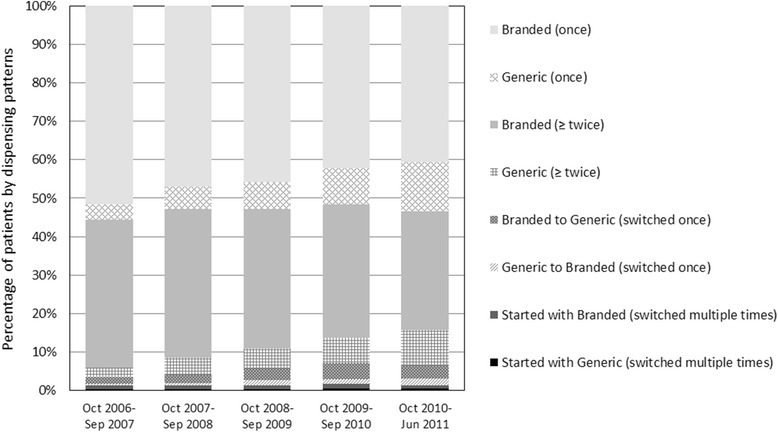
**Changes in dispensing patterns of branded and generic drugs over time.**

Dispensing patterns changed over time as shown in Figure 1. The percentage of patients who received only generic drugs (patterns 2 and 4) increased over time (6.5% at the first term to 22.1% at the last term), while that of patients who received only branded drugs (patterns 1 and 3) decreased (90.0% to 71.4%). The percentage of patients who started with branded drugs and then switched to generic drugs increased (1.8% to 3.3%).

### Prescription and dispensing procedure

**Figure 2:**
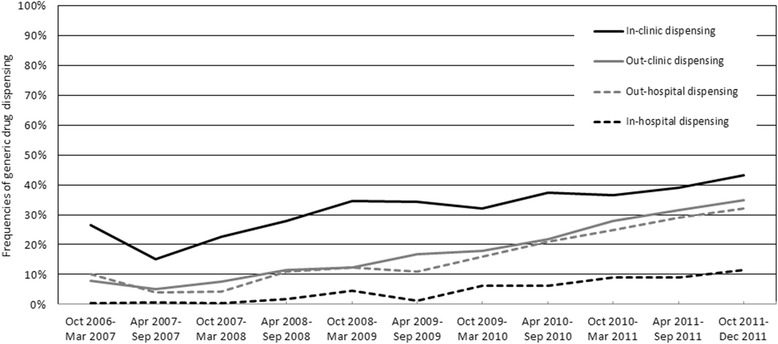
**Differences in frequencies of generic drug dispensing by prescription and dispensing settings.**

The frequency of generic drug dispensing steadily increased over time in all the four prescription and dispensing patterns (Figure 2). It was highest for in-clinic dispensing (43.3% at the last term), but lowest for in-hospital dispensing (11.5%). A similar trend was observed in out-clinic and out-hospital dispensing.

### Share trend

**Figure 3:**
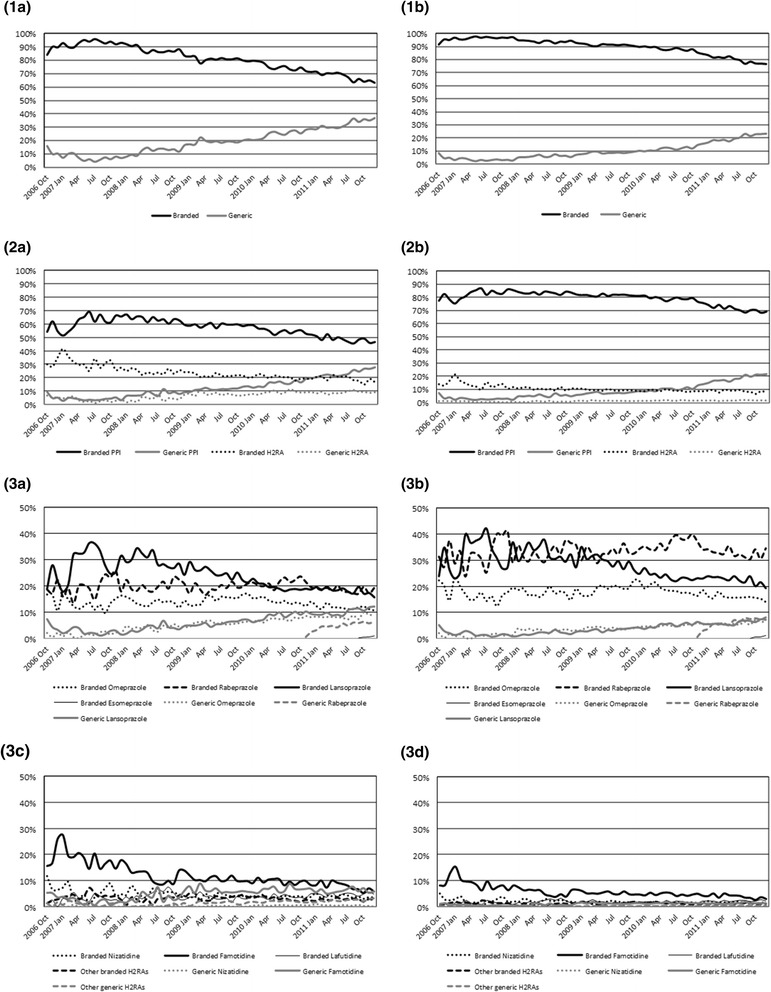
**Volume and value shares of branded and generic drugs by drug classes and molecules. (1a)** overall volume share, **(1b)** overall value share, **(2a)** volume share by drug class, **(2b)** value share by drug class, **(3a)** volume share of PPIs, **(3b)** value share of PPIs, **(3c)** volume share of H_2_RAs, **(3d)** value share of H_2_RAs.

Trends of volume and value shares are shown in Figure 3. As of December 2011, the shares of generic drugs accounted for 36.8% in volume and 23.3% in value (Figures 3-1a and 3-1b). The volume share of PPIs slightly increased due to the rise in generic PPIs (Figure 3-2a). The volume share of generic PPIs increased to a greater extent than that of generic H_2_RAs, while that of both branded PPIs and H_2_RAs decreased. The volume share of each molecule showed that generic lansoprazole, omeprazole and rabeprazole increased and branded lansoprazole and famotidine decreased (Figures 3-3a and 3-3c). The other branded and generic H_2_RAs roughly remained static in volume share over the study period and did not completely compensate for the decrease of branded famotidine. As PPIs are generally more expensive than H_2_RAs, the value share of H_2_RAs was low (Figures 3-2b, 3-3b, and 3-3d).

### Switching between branded and generic drugs

We analyzed the conditions of switching in detail using data of patients who switched from branded to generic drugs once (n = 486; pattern 5 in Table 1) and generic to branded drugs once (n = 198; pattern 6). In patients experienced switching from branded to generic drugs, the majority of patients received drugs in the same drug class (PPIs: 66.3% and, H_2_RAs: 10.3%), but remaining 23.5% changed drug class, with 71.9% of them changed from PPIs to H_2_RAs. In terms of the changes in molecules, more than half of patients (58.0%) received the same molecule. In contrast, in patients experienced switching from generic to branded drugs, 54.0% of patients received drugs in the same drug class (PPIs: 46.0% and, H_2_RAs: 8.1%), while 46.0% changed drug class, with 74.7% of them changed from H_2_RAs to PPIs. Only 19.2% received the same molecule, and switching from generic famotidine to branded drugs of lansoprazole (11.6%) and rabeprazole (12.6%), and from generic drugs of omeprazole or lansoprazole to rabeprazole (17.2%) were observed. In addition, 16.7% changed medical institutions at the time of switching from branded to generic drugs and 29.8% from generic to branded drugs.

### Factors associated with receiving generic drugs

The results of multivariate logistic regression analysis indicated that age and sex were not associated with receiving generic drugs (Table 2). Meanwhile, factors associated with receiving generic drugs included year of dispensing (adjusted OR 2.22, 95% CI 1.94 to 2.55 for 2009–11 v 2006–8), prescription and dispensing setting (OR 1.81, 95% CI 1.44 to 2.26 for prescription in hospitals and dispensing in community pharmacies; OR 2.21, 95% CI 1.80 to 2.72 for prescription in clinics and dispensing in community pharmacies; and OR 4.55, 95% CI 3.68 to 5.62 for prescription and dispensing in clinics v prescription and dispensing in hospitals) and H_2_RAs (OR 1.64, 95% CI 1.49 to 1.81 compared to PPIs).

**Table 2:** **Factors associated with receiving generic drugs at initial dispensing**

**Variables**	**Reference**	**Adjusted odds ratio (95% CI)**	**P value**
Female	Male	1.04 (0.95 to 1.13)	0.39
Age: <45 years old	≥45 years old	1.02 (0.94 to 1.11)	0.65
Dispensing: 2009–2011	2006–2008	2.22 (1.94 to 2.55)	<0.001
Out-hospital dispensing	In-hospital dispensing	1.81 (1.44 to 2.26)	<0.001
Out-clinic dispensing	In-hospital dispensing	2.21 (1.80 to 2.72)	<0.001
In-clinic dispensing	In-hospital dispensing	4.55 (3.68 to 5.62)	<0.001
H_2_RAs	PPIs	1.64 (1.49 to 1.81)	<0.001

CI: confidence interval, H_2_RAs: histamine H_2_-receptor antagonists, PPIs: proton pump inhibitors.

## Discussion

This retrospective observational study showed that branded drugs were still frequently used between 2006 and 2011 in Japan despite an increase in the share of generic drugs. We also found that generic PPIs replaced not only the corresponding branded PPIs but also the different molecules of PPIs after the introductions to the market. Changes in shares of individual molecules within a drug class have also been reported in other studies [14,15].

In addition, we demonstrated that generic PPIs might replace branded H_2_RAs in the patients with GERD. The substitution of branded H_2_RAs with generic PPIs could be related not only to government implementation of generic drugs but also to the change in approach to treating GERD. A GERD management guideline was published by the Japanese Society of Gastroenterology in November 2009, with PPIs recommended as first-line drugs [20], however, a rapid shift of dispensing from H_2_RAs to PPIs was not observed following this publication (Figure 3). We therefore speculated that studies of clinical efficacy [28] and cost-effectiveness [29-31] on PPIs, and the activities of the Society for GERD [32] had contributed to steady increase in the dispensing of PPIs. Furthermore, the affordable price, availability, and duration of marketing of generic PPIs may also have facilitated the replacement from H_2_RAs to PPIs.

The analysis of the prescription and dispensing procedure showed that the frequency of generic drug dispensing was only 11.5% in the last period of in-hospital dispensing (Figure 2). One general reason for infrequent dispensing of generic drugs might be the higher profit margin of branded drugs derived from the difference between wholesale and reimbursement prices [8,33]. However, this could not explain the disparity of the proportion of generic drugs between in-clinic and in-hospital dispensing. It is likely that other factors influence the selection of branded or generic drugs, including stubborn resistance to generic drugs, economic burdens due to the stock of various generic drugs, and the premiums for the use of generic drugs on remuneration for medical services. Further research is needed to clarify reasons why generic drugs were dispensed infrequently in in-hospital dispensing settings compared with other prescription and dispensing settings because there is more room for promoting the use of generic drugs.

Changes in dispensing patterns suggested a potentially effective approach to promoting generic drugs. The percentage of patients who received generic drugs once or multiple times from the initial dispensing increased over time, whereas the percentage of patients who switched from branded to generic drugs remained very small. Thus, starting treatment with generic drugs would be more acceptable for patients and/or medical professionals compared to switching from branded to generic drugs once treatment was initiated. Therefore, we considered that a more active approach to the use of generic drugs from the initiation of treatment onwards was needed. Our findings on the initial dispensing of GERD treatments suggested that prescribing in hospitals, especially dispensing in in-house pharmacies adversely affected the use of generic drugs. More research is needed to identify the reasons that underlie these observations.

Limitations to the present study need to be acknowledged. The claims database includes only a small proportion of people over 60 years of age. The study was biased towards a younger population than that for actual patients with GERD [34]. Treatments and selections of branded or generic drugs for GERD observed in the study may be different from those in older patients. In addition, the data available was limited to years and months in medical claims hence we compiled the data on a monthly basis. The accuracy of diagnosis of GERD written on claims could potentially have its limits since GERD was potentially diagnosed in claims in order to prescribe PPIs or H_2_RAs even if the patient is not actually suffering from GERD. We therefore attempted to remove non-GERD patients by setting strict inclusion and exclusion criteria.

The detailed analysis of the conditions at switching suggested that the treatment strategy for GERD might influence the selection of branded or generic drugs. Patients who changed the medical institutions and switched from H_2_RAs to PPIs might have uncontrolled GERD and received more intensive treatment in other medical institutions. We could not specify the reasons for switching from this study with claims data in which only a small percentage of patients experienced switching between branded and generic drugs. Further studies are required in order to clarify underlying problems in the use of generic drugs, such as dissatisfaction of patients or physicians, policy of medical institutions and pharmacies, drug stocks [35], or cultural, industrial or policy factors such as patients’ experiences with generic drugs [36], market competition [36], and drug-price margin [8] in the context of Japan.

## Conclusions

This study revealed that branded drugs for GERD were still dispensed frequently despite an increase in the share of generic drugs. Changes in the approach to treating GERD and prescription and dispensing setting also seemed to influence the dispensing of generic drugs as well as government implementation. Our findings based on generic drugs utilization in patients with the specific disease will give useful implications for physicians to increase their awareness about prescribing generic drugs and for policy makers to conduct further implementation of generic drugs with a more accurate understanding of the use of generic drugs.
